# The combined effect of metformin and mirabegron on diet‐induced obesity

**DOI:** 10.1002/mco2.207

**Published:** 2023-02-14

**Authors:** Xin‐Yuan Zhao, Ying Liu, Xuan Zhang, Ben‐Chi Zhao, George Burley, Zhi‐Can Yang, Yi Luo, An‐Qi Li, Ruo‐Xin Zhang, Zhi‐Ying Liu, Yan‐Chuan Shi, Qiao‐Ping Wang

**Affiliations:** ^1^ Laboratory of Metabolism and Aging School of Pharmaceutical Sciences (Shenzhen) Shenzhen Campus of Sun Yat‐sen University Sun Yat‐sen University Shenzhen China; ^2^ Obesity and Metabolic Disease Research Group Diabetes and Metabolism Division Garvan Institute of Medical Research Sydney New South Wales Australia; ^3^ School of Clinical Medicine St Vincent's Clinical Campus Faculty of Medicine and Health University of New South Wales Sydney New South Wales Australia

**Keywords:** β3‐adrenergic receptor agonist, combination therapy for obesity, energy expenditure, food intake, metformin, thermogenesis

## Abstract

Anti‐obesity medications act by suppressing energy intake (EI), promoting energy expenditure (EE), or both. Metformin (Met) and mirabegron (Mir) cause weight loss by targeting EI and EE, respectively. However, anti‐obesity effects during concurrent use of both have yet to be explored. In this study, we investigated the anti‐obesity effects, metabolic benefits, and underlying mechanisms of Met/Mir combination therapy in two clinically relevant contexts: the prevention model and the treatment model. In the prevention model, Met/Mir caused further 12% and 14% reductions in body weight (BW) gain induced by a high‐fat diet compared to Met or Mir alone, respectively. In the treatment model, Met/Mir additively promoted 17% BW loss in diet‐induced obese mice, which was 13% and 6% greater than Met and Mir alone, respectively. Additionally, Met/Mir improved glucose tolerance and insulin sensitivity. These benefits of Met/Mir were associated with increased EE, activated brown adipose tissue thermogenesis, and white adipose tissue browning. Significantly, Met/Mir did not cause cardiovascular dysfunction in either model. Together, the combination of Met and Mir could be a promising approach for the prevention and treatment of obesity by targeting both EI and EE simultaneously.

## Graphical abstract

The combined metformin/mirabegron treatment not only prevents the development of diet‐induced obesity but also promotes weight loss in established diet‐induced obesity. Furthermore, metformin/mirabegron treatment induces a greater reduction in body weight than either treatment when used alone. Mechanistically, these benefits on weight loss were mainly achieved by activating brown adipose tissue thermogenesis and promoting subcutaneous white adipose tissue browning, leading to enhanced energy expenditure.

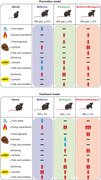

## 1 INTRODUCTION

Obesity is a rampant public health issue across the globe, affecting 671 million adults as of 2016.^1^ By 2030, approximately 1.12 billion individuals will have obesity worldwide.^2^ Obesity is a chronic condition that leads to numerous other serious diseases.^3^, ^4^, ^5^, ^6^, ^7^ The etiology and progression of obesity involve a complex multifactorial interaction of genetic, biological, and environmental components.^4^, ^8^ Long‐term energy imbalance where energy intake (EI) exceeds energy expenditure (EE) results in obesity.^9^, ^10^ Lifestyle modifications are essential to tackling obesity, but due to their difficulty and inherent limitations, they have achieved limited success in maintaining long‐term weight loss. Pharmacotherapy is therefore needed to improve the efficacy of lifestyle interventions for individuals with obesity. Since the 1900s, approximately 27 anti‐obesity drugs have been developed to modulate either EI or EE by targeting various pathways in the central nervous system or peripheral organs.^11^ Such drugs have traditionally been implemented as monotherapy, in which a single drug is given to act on one specific pathway or aspect of energy homeostasis. However, this approach has failed to achieve clinically meaningful efficacy, necessitating the development of alternative treatment regimens.^12^ Combination therapy could be a promising option. There are currently five anti‐obesity medications on the market approved for long‐term weight loss, two of which are combination therapies: phentermine–topiramate and naltrexone–bupropion.^13^ Both of these combination therapies supposedly produce synergistic effects on central pathways,^14^ suppressing food intake to induce weight loss. This is direct clinical evidence of the superiority of combination therapy in obesity treatment, necessitating further investigation into other possible drug combinations. An especially exciting prospect is the combination of peripherally acting weight‐loss drugs, or drugs that target EE as opposed to EI, which to the best of our knowledge remains an unexplored area.

Metformin (Met) is the current first‐line anti‐diabetic prescription medication worldwide.^15^ This drug lowers blood glucose levels primarily by improving insulin sensitivity in peripheral tissues, particularly the liver and skeletal muscle.^16^ It is also used by clinicians as an off‐label weight‐loss drug, as evidence has shown that it could promote weight loss via unknown mechanisms in humans overweight or with obesity.^17^, ^18^ Interestingly, recent studies have demonstrated that Met not only prevents high‐fat diet (HFD)‐induced BW gain^19^, ^20^, ^21^ but also causes weight loss in an established diet‐induced obesity (DIO) model.^22^ This effect on weight reduction appeared partially due to the suppression of feeding that was mediated by the circulating growth/differentiation factor 15 (GDF15) and its receptor GDNF family receptor α‐like (GFRAL) in the hindbrain.^22^ However, the mechanism by which Met modulates EE in peripheral tissues, if at all, remains unclear.

Brown adipose tissue (BAT) and beige white adipose tissue (WAT) have been increasingly recognized as critical regulators of whole‐body metabolism and EE and are considered promising targets for anti‐obesity therapeutics. BAT is enriched with mitochondria in which uncoupling protein 1 (UCP1) is highly expressed. UCP1 dissipates excess energy as heat in a process known as thermogenesis.^23^ Activation of BAT and promotion of WAT browning can be induced by cold exposure or sympathetic nerve innervation via the β3‐adrenergic receptor (β3‐AR), which is abundantly expressed in adipose tissues, particularly BAT.^24^, ^25^ Mirabegron (Mir), a β3‐AR agonist approved for treating overactive bladder syndrome,^26^ promotes BAT thermogenesis in mice^27^ and humans.^28^, ^29^, ^30^ Therefore, Mir is a promising candidate drug to promote weight loss by boosting EE. However, it is unknown whether Mir will remain effective in enhancing EE when EI is suppressed, such as in Met treatment, and whether additive or synergistic effects on weight loss will be achieved by combining these two drugs.

In this study, we used Met as an EI suppressant and Mir as an EE booster to investigate the combined effects of Met/Mir in preventing obesity development as well as in treating established DIO in mice. We measured several metabolic parameters and investigated the underlying molecular mechanisms, with a special focus on pathways involved in thermogenesis. We also evaluated the safety of this combination treatment, especially on potential cardiovascular side effects. Our results showed that Met/Mir produced additive effects on the prevention and treatment of obesity compared with monotherapy with either drug, with minimal side effects observed.

## 2 RESULTS

### 2.1 Met/Mir has an additive effect on preventing HFD‐induced weight and fat gain in mice

**FIGURE 1:**
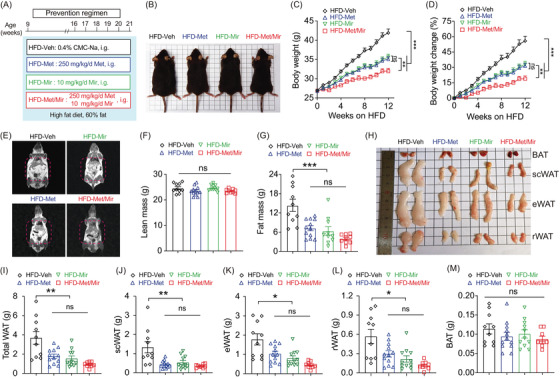
Metformin (Met)/mirabegron (Mir) has an additive effect on preventing weight gain in the prevention model. (A) The schematic diagram of the experimental design. Mice were fed a high‐fat diet (HFD) and concurrently administered with vehicle (Veh), Met, Mir, or Met/Mir at 5:00 p.m. daily by gavage for 12 weeks. (B–H) The representative mice (B), body weight (BW) (C), BW change (D), representative nuclear magnetic resonance (NMR) images (E), lean mass (F), and fat mass (G) are shown in HFD‐fed mice after treatments for 12 weeks. (H–M) The representative images (H) and wet tissue weight of total white adipose tissue (WAT) (I), subcutaneous WAT (scWAT) (J), epidydimal WAT (eWAT) (K), retroperitoneal WAT (rWAT) (L), and brown adipose tissue (BAT) (M) are shown in HFD‐fed mice after treatment for 12 weeks (*n* = 9–12). Data are reported as mean ± standard error of mean (S.E.M.). One‐way analysis of variance (ANOVA) with Tukey's multiple comparisons test (F, G, I–M) and two‐way ANOVA with Sidak multiple comparisons test (C, D) were used. n.s., not significant, ^*^ *p* < 0.05, ^**^ *p* < 0.01, and ^***^ *p* < 0.001

Mice were fed a HFD and simultaneously received daily gavage with vehicle (Veh), Met, Mir, or Met/Mir for 12 weeks (Figure 1A). The body weight (BW) of drug‐treated mice was significantly lower than that of Veh‐treated mice under HFD feeding (Figure 1B). HFD‐fed mice treated with Met or Mir alone showed 24% (6.8 g) or 23% (6.3 g) less BW gain, respectively, when compared to the Veh‐treated mice. Importantly, Met/Mir‐treated mice displayed 37% less BW gain, which was a further 12% (3.2 g) and 14% (3.6 g) reduction in weight gain when compared to either Met or Mir alone (Figure 1C,D). This result suggests that Met/Mir has a combined effect in preventing weight gain in mice on HFD feeding.

Next, we measured the body composition using nuclear magnetic resonance (NMR) (Figure 1E). No drug treatment altered lean mass (Figure 1F), but Met and Mir mice had 49% (7.1 g) and 56% (8.0 g) less fat mass than Veh mice (14.4 g), respectively. Met/Mir treatment markedly reduced fat mass by 72% (10.4 g), although this reduction was not significant compared to Met and Mir mono‐treatment (Figure 1G). These results were further confirmed by the dissected weights of major white fat depots (subcutaneous, epidydimal, and retroperitoneal) (Figure 1H,I). In particular, Met, Mir, and Met/Mir treatments considerably decreased subcutaneous WAT (scWAT) by 0.89 g (–67%), 0.80 g (–60%), and 0.98 g (–73%), respectively (Figure 1J). Despite a lack of statistical significance, Met/Mir treatment produced a greater reduction in fat mass than monotherapy. The Met, Mir, and Met/Mir treatments reduced the epidydimal WAT (eWAT) weight by 0.74 g (–42%), 0.98 g (–55%), and 1.32 g (74%), respectively, when compared to the Veh treatment (Figure 1K). Similar results were observed in retroperitoneal WAT (rWAT), which was reduced by 0.27 g (–48%), 0.35 g (–62%), and 0.45 g (–80%) in Met, Mir, and Met/Mir mice, respectively, compared to Veh mice (Figure 1L). Of note, there was a non‐significant decrease in BAT weight in Met, Mir, or Met/Mir mice compared to Veh mice (Figure 1M). Taken together, Met/Mir prevents BW gain in an additive manner in HFD‐fed mice, which is mainly caused by a lower fat mass.

### 2.2 Met/Mir has no impact on food intake but increases EE in HFD‐fed mice

**FIGURE 2:**
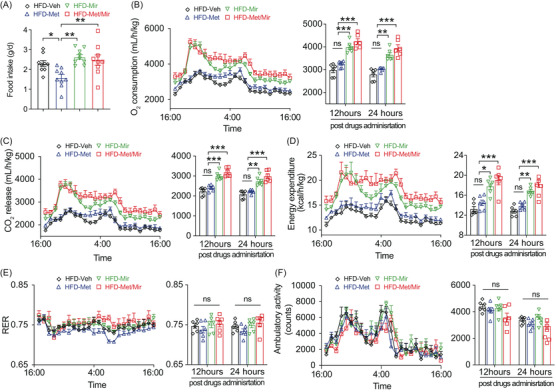
Metformin (Met)/mirabegron (Mir) exerts a combined effect on energy expenditure (EE) in the prevention model. (A) Food intake in mice fed a high‐fat diet (HFD) and simultaneously treated with vehicle (Veh), Met, Mir, or Met/Mir for 12 weeks (*n* = 9). (B–F) The O_2_ consumption (B), CO_2_ release (C), EE (D), respiratory exchange ratio (RER) (E), and ambulatory activity (F) were measured for 24 h in HFD‐fed mice after treatment for 12 weeks (*n* = 6). All drugs were administered at 5:00 p.m. Data are reported as mean ± standard error of mean (S.E.M.). One‐way analysis of variance (ANOVA) with Tukey's multiple comparisons test was used. n.s., not significant, ^*^ *p* < 0.05, ^**^ *p* < 0.01, and ^***^ *p* < 0.001

Since obesity occurs under a long‐term energy imbalance, we next evaluated the effects of Met/Mir on EI and EE. Consistent with previous reports,^19^, ^31^ Met decreased food intake, while Mir had no impact on food intake compared to Veh (Figure 2A). Interestingly, Met's appetite‐suppressing effect was abolished when Met was combined with Mir (Figure 2A). Notably, no difference in water consumption was observed across all groups (Figures S1). These findings indicate that food intake is unlikely to be responsible for the reduced BW gain from the Met/Mir treatment.

We next subjected mice to metabolic cages to measure EE after 12 weeks of treatment under ad libitum HFD feeding. When compared to Veh, Mir, or Met/Mir caused significantly elevated O_2_ consumption, CO_2_ release, and EE when data were expressed as an hourly average and as a 12‐ or 24‐h average post‐drug administration (Figure 2B–D). Noticeably, Met/Mir mice had the highest O_2_ consumption, CO_2_ release, and EE among all groups (Figure 2B–D). This suggests that Mir has a dominant effect on EE when used with Met. The respiratory exchange ratio (RER), an indicator of metabolic fuel preference, displayed no difference among all groups (Figure 2E). In addition, no difference in physical activity was observed among all groups (Figure 2F).

Met/Mir mice displayed a significant increase in food intake and EE when compared to their Met counterparts (Figure 2A). To determine whether this elevated EE was caused by food‐induced thermogenesis that was associated with increased food intake, we performed a 2‐week HFD pair‐feeding experiment in a separate cohort of wild‐type mice where an equal amount of food was given to all the mice. Met caused no change in O_2_ consumption, CO_2_ release or EE, whereas Mir caused an elevation in O_2_ consumption, CO_2_ release, and EE (Figure S2A–C). When compared to Veh or Met mice, both Mir and Met/Mir mice displayed a non‐significant increase in O_2_ consumption, CO_2_ release, and EE within 6 h post‐drug administration (Figure S2A–C). Interestingly, only Met/Mir significantly decreased the RER (Figure S2D) suggesting a strong promotion of lipid oxidation. No difference in physical activity was observed among the groups (Figure S2E). All findings clearly demonstrate that the enhanced EE in the Met/Mir treatment is independent of increased food intake.

### 2.3 Met/Mir has an additive effect on improving insulin responsiveness in HFD‐fed mice

**FIGURE 3:**
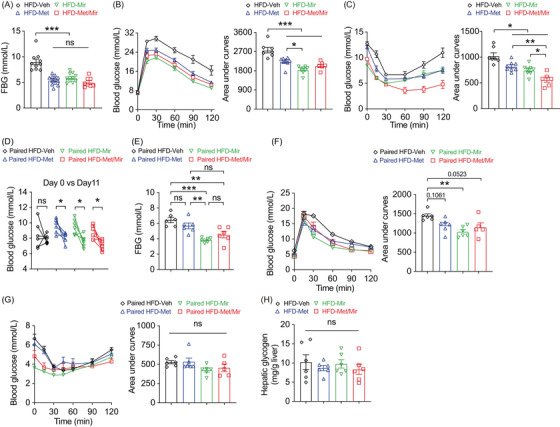
Metformin (Met)/mirabegron (Mir) has an additive effect on improving insulin responsiveness in the prevention model. (A–C) Fasting blood glucose (FBG) (A), intraperitoneal glucose tolerance test (IPGTT) (B), and insulin tolerance test (ITT) (C) in high‐fat diet (HFD)‐fed mice after treatments for 12 weeks (*n* = 9–12), 10 weeks (*n* = 6–8), and 12 weeks (*n* = 6–8), respectively. (D–G) Blood glucose (D), 6‐h FBG (E), IPGTT (F), and ITT (G) in HFD pair‐feeding mice after treatments for 11, 8, 8, and 10 days, respectively (*n* = 5–6). (H) Hepatic glycogen content in HFD‐fed mice after 12 weeks treatment (*n* = 6). Data are reported as mean ± standard error of mean (S.E.M.). One‐way analysis of variance (ANOVA) with Tukey's multiple comparisons test (A–C, E–H) and paired *t*‐test with two‐tailed test were used (D). n.s., not significant, ^*^ *p* < 0.05, ^**^ *p* < 0.01, and ^***^ *p* < 0.001

Previous studies have shown that either Met or Mir is effective in improving glucose homeostasis and insulin responsiveness.^27^, ^28^, ^32^ We therefore investigated whether Met/Mir has an additive effect on glucose metabolism. Elevated fasting blood glucose (FBG) is an indicator of prediabetic conditions.^33^ As expected, compared to Veh, Met, and Mir alone significantly decreased FBG; however, Met/Mir did not produce an additional effect on FBG (Figure 3A). Met or Mir alone significantly improved glucose tolerance, as observed by lower blood glucose excursion and confirmed by the area under the curve (AUC), yet no further improvement was seen by Met/Mir treatment (Figure 3B). Interestingly, while all drug‐treated mice displayed increased insulin responsiveness, Met/Mir mice had a further improvement when compared to monotherapy mice (Figures 3C and S3). These results suggest that Met/Mir improves in vivo insulin responsiveness under ad libitum feeding.

We next asked whether the improved glucose tolerance was a direct effect of Met treatment and not a flow‐on effect of reduced food intake observed in Figure 2A. We assessed glucose metabolism in a cohort of mice after a 2‐week period of HFD pair‐feeding and drug treatment. Veh mice did not have HFD‐induced elevation in blood glucose due to limited HFD consumption. Met and Mir monotherapy reduced basal blood glucose on day 11, and a similar reduction was observed under Met/Mir treatment (Figure 3D). Under pair‐feeding, Met did not alter 6‐h FBG levels, while Mir and Met/Mir significantly reduced them, but no further difference was observed under Met/Mir compared to either Met or Mir alone (Figure 3E). When compared to Veh, Met slightly improved glucose tolerance, while Mir significantly enhanced glucose tolerance, and Met/Mir caused a strong trend toward an improvement in glucose tolerance (*p* = 0.0523) (Figure 3F). No effect on insulin responsiveness was observed among all groups (Figure 3G). Additionally, after all treatments, hepatic glycogen content was not altered by Met, Mir, or Met/Mir (Figure 3H). Collectively, these data confirm that the effect of Met/Mir on improved glycemic control is independent of food intake.

### 2.4 Met/Mir has an additive effect on lipolysis and fatty acid oxidation in BAT in HFD‐fed mice

**FIGURE 4:**
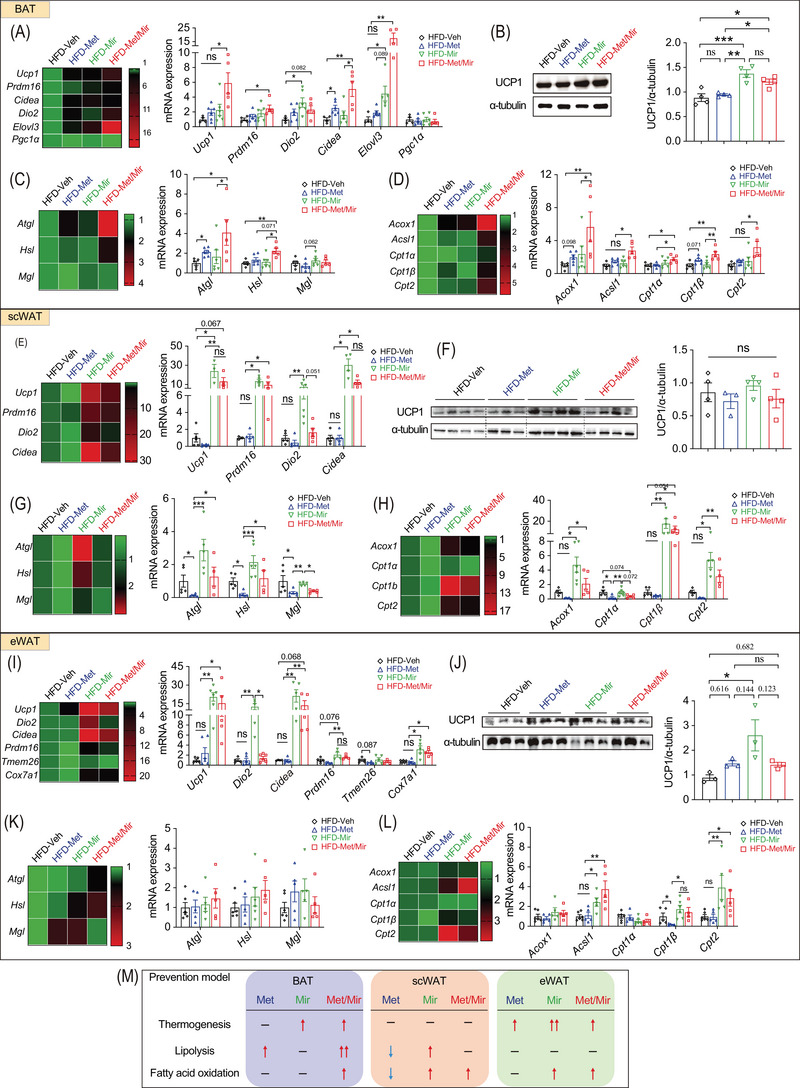
Metformin (Met)/mirabegron (Mir) increases lipolysis and fatty acid oxidation and activates brown adipose tissue (BAT) thermogenesis. Mice were fed a high‐fat diet (HFD) and simultaneously treated with vehicle (Veh), Met, Mir, or Met/Mir for 12 weeks. (A–D) The mRNA expression of thermogenic genes (A, *n* = 5–6) and uncoupling protein 1 (UCP1) protein (B, *n* = 4), lipolytic genes (C, *n* = 5–6), and genes involved in fatty acid oxidation (D, *n* = 5–6) in BAT. (E–H) The mRNA expression of thermogenic genes (E, *n* = 4–6), UCP1 protein (F, *n* = 4), lipolytic genes (G, *n* = 4–6), and genes involved in fatty acid oxidation (H, *n* = 5–6) in subcutaneous white adipose tissue (scWAT). (I–L) The mRNA expression of thermogenic genes (I, *n* = 5–6), UCP1 protein (J, *n* = 3), lipolytic genes (K, *n* = 5–6), and fatty acid oxidation genes (L, *n* = 5–6) in epidydimal WAT (eWAT). (M) In the prevention model, a summary of changes in thermogenesis, lipolysis, and fatty acid oxidation in BAT, scWAT, and eWAT after Met and/or Mir treatment. Data are reported as mean ± standard error of mean (S.E.M.). Kruskal–Wallis test with uncorrected Dunn's test (A, C–E, G–I, K–L) and one‐way analysis of variance (ANOVA) with Tukey's multiple comparisons test (B, F, J) were used. n.s., not significant, ^*^ *p* < 0.05, ^**^ *p* < 0.01, and ^***^ *p* < 0.001

To gain insight into how Met/Mir generates metabolic benefits under HFD feeding, we assessed the expression of key genes involved in thermogenesis, lipolysis, and fatty acid oxidation in BAT and WAT. In BAT, both Met alone and Mir alone caused a non‐significant trend of increased *Ucp1* mRNA expression, while Met/Mir significantly upregulated its expression (Figure 4A), which was confirmed by UCP1 protein levels (Figure 4B). For other thermogenic markers, when compared to Veh, Met increased the mRNA expression of *Cidea*, and Mir increased that of *Elovl3*; Met/Mir significantly upregulated the mRNA levels of *Prdm16*, *Dio2*, *Cidea*, and *Elovl3* (Figure 4A). Consistent with the observed enhancement of thermogenesis, Met/Mir significantly elevated the expression of *Atgl* and *Hsl*, two key regulators in lipolysis (Figure 4C), and a panel of markers involved in mitochondrial fatty acid oxidation, including *Acox1*, *Acsl1*, *Cpt1α*, *Cpt1β*, and *Cpt2* (Figure 4D). These results suggest that Met/Mir treatment additively increases lipolysis to provide fuel to the mitochondria, thereby boosting fatty acid oxidation and enhancing thermogenesis.

Browning of WAT, especially subcutaneous fat depots, increases thermogenesis and EE and improves glucose tolerance. We evaluated the expression of key beige fat markers in scWAT. Compared to Veh, Met caused a non‐significant decrease in *Ucp1* mRNA (Figure 4E) and protein levels (Figure 4F), whereas Mir significantly increased the mRNA levels of *Ucp1*, *Prdm16*, *Dio2*, and *Cidea* (Figure 4E). The mRNA expression of these genes was comparable between Mir and Met/Mir (Figure 4E). However, in contrast to mRNA levels, UCP1 protein levels were not significantly altered (Figure 4F). Consistent with previous reports,^34^ Met decreased the mRNA expression of lipolytic genes in scWAT (Figure 4G). *Cpt1α*, encoding a rate‐limiting enzyme that transports fatty acids into mitochondria for β‐oxidation,^35^ was also downregulated while other fatty acid oxidation genes were unaffected (Figure 4H). In eWAT, Mir alone and Met/Mir significantly boosted the mRNA levels of several markers involved in WAT browning, including *Ucp1*, *Dio2*, *Cidea*, *Ppdm16*, and *Cox7a1* (Figure 4I). However, when compared to the Veh group, UCP1 protein levels in all three drug treatment groups were slightly increased, but there was no difference between drug treatment groups (Figure 4J). Lipolysis was unaffected by any drug treatment (Figure 4K). Regarding fatty acid oxidation, Mir upregulated the mRNA expression of *Acsl1*, *Cpt1β*, and *Cpt2*, suggesting a possible boost in mitochondrial activity by Mir (Figure 4L). Similar to Mir, Met/Mir increased the mRNA levels of markers involved in browning and fatty acid oxidation, while no additive effects were observed (Figure 4K,L).

Collectively, these results have demonstrated a fat depot‐specific response to drug treatments (Figure 4M). The additive effect of Met/Mir was more pronounced in BAT than in scWAT or eWAT. The enhanced BAT thermogenesis and browning of WAT, accompanied by improved lipolysis and fatty acid oxidation, may contribute to the observed metabolic benefits in Met/Mir‐treated mice.

### 2.5 Met/Mir has an additive effect on weight and fat loss in DIO mice

**FIGURE 5:**
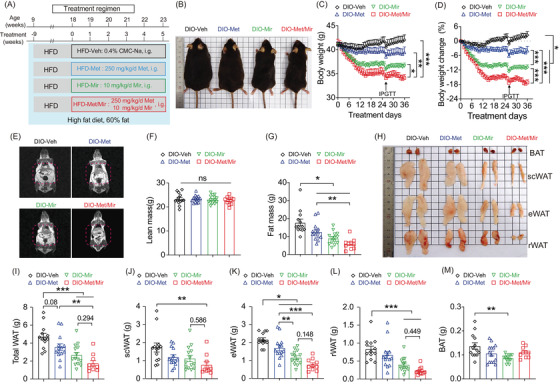
Metformin (Met)/mirabegron (Mir) has an additive effect on weight loss and white adipose tissue (WAT) reduction in the treatment model. (A) The schematic diagram of the experimental design. Mice were first fed a high‐fat diet (HFD) for 9 weeks to establish a diet‐induced obesity (DIO) phenotype (DIO mice), then the DIO mice were fed a HFD and administered with vehicle (Veh), Met, Mir, and Met/Mir at 5:00 p.m. daily by gavage for another 5 weeks. (B–H) The representative mice (B), body weight (BW) (C), BW change (D), representative nuclear magnetic resonance (NMR) images (E), lean mass (F), and fat mass (G) are shown in DIO mice after treatments. (H–N) The representative images (H) and wet weight of total WAT (I), subcutaneous WAT (scWAT) (J), epidydimal WAT (eWAT) (K), retroperitoneal WAT (rWAT) (L), and brown adipose tissue (BAT) (M) are shown in DIO mice after treatment (*n* = 10–14). Data are reported as mean ± standard error of mean (S.E.M.). One‐way analysis of variance (ANOVA) with Tukey's multiple comparisons test (F, G, I–M) and two‐way ANOVA with Sidak multiple comparisons test from 24th to 36th day in (C), and from 0th to 36th day in (D) were used. n.s., not significant, ^*^ *p* < 0.05, ^**^ *p* < 0.01, and ^***^ *p* < 0.001

We observed the combined effects of Met/Mir in preventing the development of obesity. Considering that Met is effective in treating individuals with overweight and obesity,^18^ and that clinical trials have demonstrated metabolic benefits of Mir in humans,^29^ we sought to investigate whether Met/Mir would produce more pronounced benefits on weight loss and improve metabolism in a more clinically relevant setting, such as DIO. For this, wild‐type mice were first fed a HFD for 9 weeks to establish an obese phenotype. They were subsequently divided into groups to receive Veh (DIO‐Veh), Met (DIO‐Met), Mir (DIO‐Mir), and Met/Mir (DIO‐Met/Mir) via daily oral gavage for another 5 weeks with HFD feeding continued (Figure 5A). At the end of treatment, compared to the Veh mice that continued to gain BW as expected, all three drug‐treated DIO mice displayed significant weight loss (Figure 5B–D). BW in DIO‐Met and DIO‐Mir mice was significantly reduced by 4% (–1.57 g) and 11% (–4.44 g), respectively (Figure 5C,D). Strikingly, Met/Mir induced a 17% (–7.13 g) weight loss, which was significantly greater than that induced by Met or Mir alone (Figure 5C,D). This result is suggestive of an additive effect of Met/Mir on promoting weight loss in DIO mice.

The body composition of the mice was determined using NMR (Figure 5E). Lean mass was not affected by any drug treatment (Figure 5F). Compared to DIO‐Veh mice, fat mass in DIO‐Met and DIO‐Mir mice was significantly reduced by 29% (–5.11 g) and 49% (–8.70 g), respectively (Figure 5G). Met/Mir considerably reduced fat mass in DIO mice by 67% (–11.93 g), which was significantly more than that by Met or Mir alone (Figure 5G). The fat loss was further confirmed by the dissected weights of the three major fat depots (Figure 5H,I). Met caused a strong trend toward a reduction in total WATs (25%, –1.16 g, *p* = 0.08), while Mir and Met/Mir significantly reduced total WAT weight by 43% (–2.05 g) and 63% (–2.96 g), respectively (Figure 5I). Met, Mir, and Met/Mir decreased scWAT weight by 31% (–0.53 g), 35% (–0.61 g), or 56% (–0.96 g), respectively, although only the reduction by Met/Mir reached statistical significance when compared to Veh (Figure 5J). This finding implies that Met/Mir has a combined effect on scWAT loss. Moreover, Met, Mir, and Met/Mir significantly reduced eWAT weight by 0.46 g (–21%), 1.00 g (–46%), and 1.39 g (–64%), respectively. Both Mir and Met/Mir strongly decreased eWAT weight compared to Met monotherapy, indicating a predominant effect of Mir in the combination treatment (Figure 5K). Less pronounced effects were seen in rWAT, with a marked reduction seen in the Mir and Met/Mir groups without a statistical difference between them (Figure 5L). Notably, BAT weight was significantly reduced in DIO‐Mir mice but not in DIO‐Met or Met/Mir‐DIO mice (Figure 5M). In summary, Met/Mir has an additive effect on weight loss in DIO mice, which is associated with significant fat loss, especially in scWAT.

### 2.6 Met/Mir exerts an additive effect on EE in DIO mice

**FIGURE 6:**
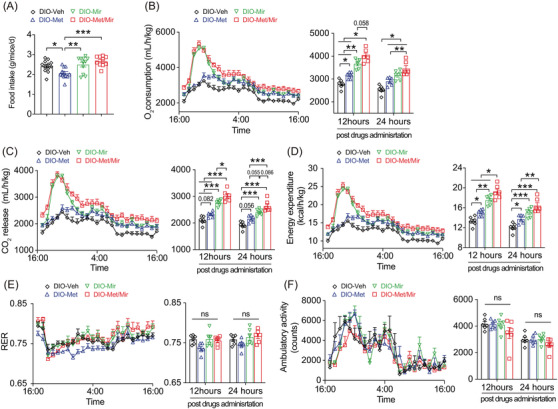
Metformin (Met)/mirabegron (Mir) exerts an additive effect on energy expenditure (EE) in diet‐induced obesity (DIO) mice in the treatment model. (A) Food intake in DIO mice after being treated with vehicle (Veh), Met, Mir, or Met/Mir for 5 weeks (*n* = 10–12). (B–F) The O_2_ consumption (B), CO_2_ release (C), EE (D), respiratory exchange ratio (RER) (E), and ambulatory activity (F) were measured for 24 h in DIO mice after being treated with Veh, Met, Mir, or Met/Mir treatment for 5 weeks (*n* = 6). All drugs were administered at 5:00 p.m. every day. Data are reported as mean ± standard error of mean (S.E.M.). One‐way analysis of variance (ANOVA) with Tukey's multiple comparisons test. n.s., not significant, ^*^ *p* < 0.05, ^**^ *p* < 0.01, and ^***^ *p* < 0.001

To understand the combined effect on weight loss and WAT reduction in DIO‐Met/Mir mice, we then assessed EI and EE. No differences in water intake were observed across all groups (Figure S4). Food consumption was reduced in DIO‐Met mice but was unchanged in DIO‐Mir and DIO‐Met/Mir mice when compared to their Veh counterparts (Figure 6A). Therefore, the EI is not responsible for the observed pronounced weight loss in DIO‐Met/Mir mice. Previous studies have shown that Met and Mir alone can enhance EE in DIO mice.^22^, ^27^ To determine whether Met/Mir has a combined effect on EE, we subjected DIO mice to metabolic cages after 5 weeks of drug treatments. As expected, Met increased O_2_ consumption and CO_2_ release in DIO mice post‐drug administration (Figure 6B,C). As a result, EE was increased in DIO‐Met mice (Figure 6D). Similarly, in DIO‐Mir mice, a significant increase in O_2_ consumption, CO_2_ release, and EE was observed (Figure 6B–D). Strikingly, Met/Mir boosted EE to the highest of the four treatment groups, significantly higher than that induced by Met or Mir alone (Figure 6D). Neither RER nor physical activity was altered by any treatment (Figure 6E,F). Together, these data strongly suggest that the additive effect of Met/Mir on enhancing EE is a major contributor to the reduced BW and fat mass in DIO mice.

### 2.7 Met/Mir has no combined effect on glucose homeostasis in DIO mice

**FIGURE 7:**
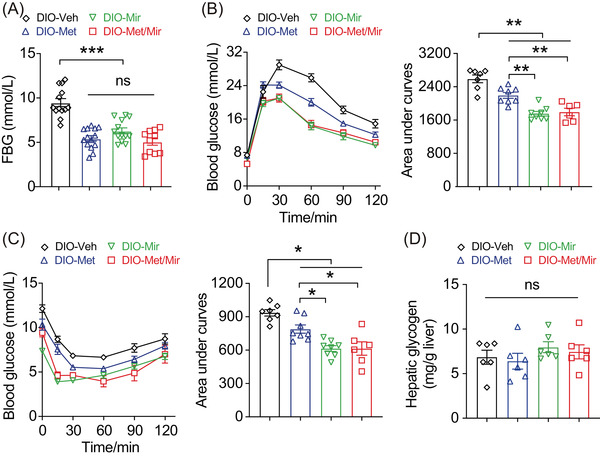
Metformin (Met)/mirabegron (Mir) improves glucose metabolism in diet‐induced obesity (DIO) mice. (A–D) Fasting blood glucose (FBG) (A), intraperitoneal glucose tolerance test (IPGTT) (B), insulin tolerance test (ITT) (C), and hepatic glycogen content (D) in DIO mice after treatments for 5 weeks (*n* = 10–14), 26 days (*n* = 6–8), 34 days (*n* = 6–8), and 5 weeks (*n* = 6). Data are reported as mean ± standard error of mean (S.E.M.). One‐way analysis of variance (ANOVA) with Tukey's multiple comparisons test was used. n.s., not significant, ^*^ *p* < 0.05, ^**^ *p* < 0.01, and ^***^ *p* < 0.001

In humans, a modest 5% weight loss is sufficient to generate clinically measurable metabolic benefits including improved fasting glycemic level and glucose homeostasis.^36^ At the end of the 5‐week treatment, all drug‐treated mice displayed significantly lower FBG levels compared to the DIO‐Veh counterparts, with no further reduction seen in DIO‐Met/Mir mice (Figure 7A). Consistent with previous reports, Met or Mir alone greatly improved glucose tolerance (Figure 7B) and insulin responsiveness (Figure 7C). Met/Mir treatment did not show an additive effect when compared with either Met or Mir alone (Figure 7B,C). Again, hepatic glycogen content was unaffected by any drug treatment in the treatment model (Figure 7D). Taken together, Met/Mir has no additional benefits in improving glucose homeostasis in DIO mice compared to either Met or Mir alone.

### 2.8 Met/Mir has a combined effect on BAT thermogenesis and scWAT browning in DIO mice

**FIGURE 8:**
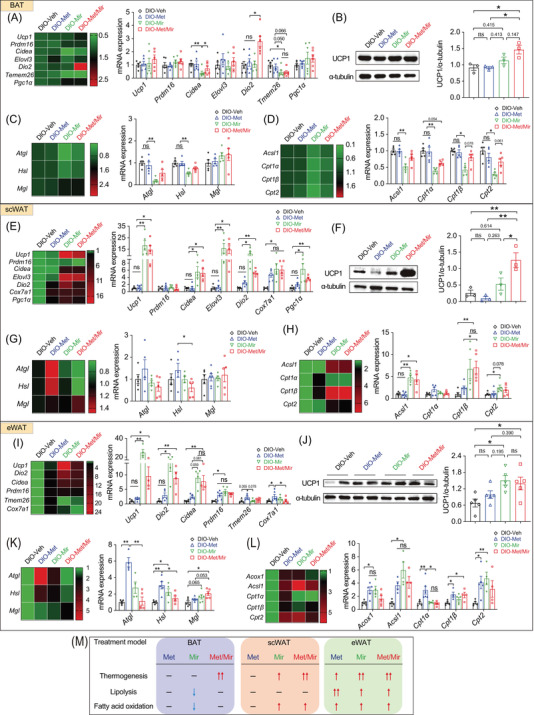
Metformin (Met)/mirabegron (Mir) has additive effects on brown adipose tissue (BAT) thermogenesis, fatty acid oxidation, and subcutaneous white adipose tissue (scWAT) browning in diet‐induced obesity (DIO) mice. DIO mice were treated with vehicle (Veh), Met, Mir, or Met/Mir for 5 weeks. (A and B) The mRNA expression of thermogenic genes (A, *n* = 5–6), uncoupling protein 1 (UCP1) protein (B, *n* = 3), lipolytic genes (C, *n* = 5–6), and genes involved in fatty acid oxidation (D, *n* = 5–6) in BAT. (E–H) The expression of thermogenic genes (E, *n* = 5–6), UCP1 protein (F, *n* = 3), lipolytic genes (G, *n* = 5–6), and genes involved in fatty acid oxidation (H, *n* = 5–6) in scWAT. (I–L) The expression of thermogenic genes (I, *n* = 5–6), UCP1 protein (J, *n* = 5), lipolytic genes (K, *n* = 5–6), genes involved in fatty acid oxidation (L, *n* = 5–6) in epidydimal WAT (eWAT). (M) A summary of thermogenesis, lipolysis, and fatty acid oxidation in BAT, scWAT, and eWAT after Met and/or Mir treatment in the treatment model. Data are reported as mean ± standard error of mean (S.E.M.). Kruskal–Wallis test with uncorrected Dunn's test (A, C–E, G–I, K–L) and one‐way analysis of variance (ANOVA) with Tukey's multiple comparisons test (B, F, and J) were used. n.s., not significant, ^*^ *p* < 0.05, ^**^ *p* < 0.01, and ^***^ *p* < 0.001

To dissect how Met/Mir additively promoted weight and fat loss in DIO mice, we evaluated the expression of some key genes involved in thermogenesis, lipolysis, and fatty acid oxidation in BAT and WAT. In BAT, the mRNA expression of thermogenic genes was generally unaffected except that Met/Mir markedly increased *Dio2* expression (Figure 8A). Despite no alteration in *Ucp1* mRNA expression (Figure 8A), UCP1 protein levels were significantly upregulated under Met/Mir treatment compared to Veh or Met treatment (Figure 8B). This indicates that Met/Mir has a combined effect in activating BAT thermogenesis at the protein level. Met and Met/Mir did not affect the expression of genes important in lipolysis (Figure 8C) or genes involved in mitochondrial fatty acid oxidation (Figure 8D). However, the mRNA expression of these genes, except for *Mgl*, was significantly downregulated by Mir (Figure 8D).

In scWAT, Met showed little impact on thermogenesis, lipolysis, and fatty acid oxidation, except for upregulating *Cox7a1* mRNA expression (Figure 8E–H). As expected, Mir strongly promoted browning in scWAT by enhancing the mRNA expression of brown fat‐specific genes including *Ucp1*, *Cidea*, *Elovl3*, *Dio2*, *Cox7a1*, and *Pgc1α* (Figure 8E). This was accompanied by increased mitochondrial fatty acid oxidation, as evidenced by increased expression of *Acsl1*, *Cpt1β*, and *Cpt2* (Figure 8H). Met/Mir led to a similar expression of key genes in WAT browning, lipolysis, and fatty acid oxidation when compared to Mir (Figure 8E–H). Strikingly, similar to that in BAT, Met/Mir additively increased UCP1 proteins in scWAT (Figure 8F), which may contribute to enhanced scWAT browning and elevated whole‐body EE.

The eWAT weight was reduced the most out of all the WAT depots, as shown in Figure 5K. Met induced thermogenesis in eWAT by elevating a selection of markers, including *Tmem26* and *Cox7a1* (Figure 8I). Strongly upregulated expression was observed in lipolytic genes, such as *Atgl* and *Hsl*, and key genes of fatty acid oxidation, including *Acox1*, *Acsl1*, *Cpt1α*, *Cpt1β*, and *Cpt2*, suggesting that an increase in fuel availability boosts mitochondrial biogenesis (Figure 8I–L). These data suggest that, unlike the limited changes in scWAT, eWAT is more responsive to Met treatment. Mir‐treated eWAT displayed upregulated mRNA expression levels in panels of markers in thermogenesis, lipolysis, and fatty acid oxidation (Figure 8I–L). Of note, unlike Met, Mir also increased UCP1 protein levels (Figure 8J), demonstrating an improved thermogenic capacity in eWAT. Met/Mir showed similar but less pronounced effects on the genes assessed compared to Mir alone (Figure 8I–L), indicating no additive or antagonizing effects of Met and Mir in modulating eWAT under DIO conditions.

Overall, Met/Mir treatment has an additive effect on BAT thermogenesis and scWAT browning but not on lipolysis, lipid oxidation, and browning in eWAT in DIO mice. These major findings are summarized in Figure 8M.

### 2.9 No cardiovascular side effects observed by Met/Mir treatment

Growing evidence has supported Met's cardioprotective effects,^37^ while the safety of Mir is still under investigation with a major concern that Mir may cause undesired side effects through nonspecific binding to other adrenergic receptors in the heart.^38^ We therefore assessed the function of the cardiovascular system in mice after treatment. In the prevention model, Met and/or Mir did not affect heart rate, systolic blood pressure (SBP), diastolic blood pressure (DBP), or mean blood pressure (MBP) (Figure S5A–D). The double product, an index of myocardial oxygen consumption, was not altered among groups (Figure S5E). Moreover, Met/Mir did not alter the weight of the liver, heart, lungs, spleen, or kidneys (Figure S5F,G) after chronic treatment under ad libitum HFD feeding. We also evaluated the safety of Met/Mir in the treatment model. Heart rate, MBP, and DBP (Figure S5H–J) were not affected by any treatment. When compared to the Veh, mono‐treatment with Met or Mir significantly increased SBP, yet Met/Mir treatment had the similar level to the Veh control (Figure S5K). Only Met caused a significant increase in the double product, while no change was observed after Mir or Met/Mir treatment (Figure S5L). Additionally, the weights of the liver, heart, lung, spleen, and kidneys in DIO mice remained unchanged (Figure S5M,N). These data suggest that in both prevention and treatment models, Met/Mir induces no obvious side effects on cardiovascular function or alteration to the weights of other important organs and tissues.

## 3 DISCUSSION

**FIGURE 9:**
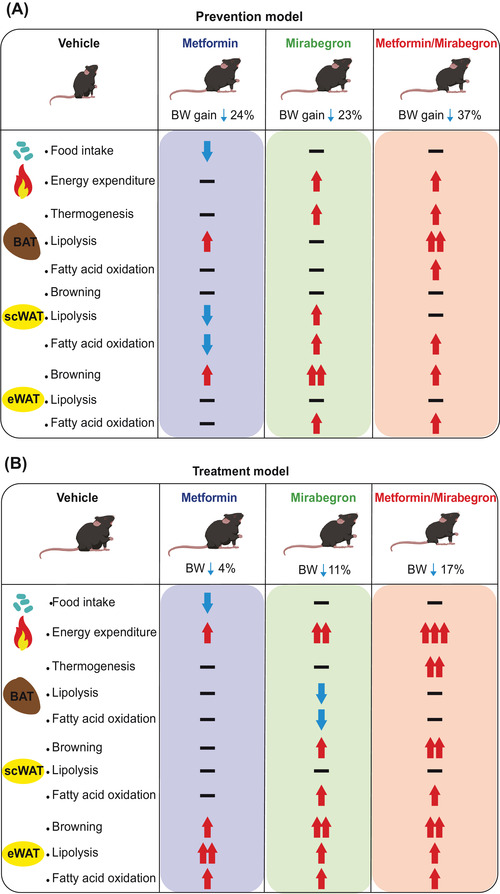
A diagram summarizing the combined effects of metformin (Met)/mirabegron (Mir) on obesity in the prevention and treatment models. (A) In the prevention model where a high‐fat diet (HFD) and drugs were administered simultaneously, Met/Mir treatment lowered weight gain in an additive manner. This is primarily due to an improvement in energy expenditure (EE) that was accompanied by upregulated expression of critical markers in lipolysis, fatty acid oxidation, and thermogenesis in brown adipose tissue (BAT). (B) In the treatment model, a diet‐induced obesity (DIO) phenotype was first established followed by 5 weeks of therapeutic treatments with Met and/or Mir. Met/Mir treatment caused marked weight loss, resulting from augmented EE

The efficacy of current anti‐obesity monotherapy has been unsatisfactory. Combination therapy, particularly one that targets both EI and EE, may be better in managing obesity and its comorbidities. In this study, we have demonstrated that the combination of Met and Mir prevents HFD‐induced obesity development in an additive manner, and promotes weight loss in established DIO. These effects on BW were mainly achieved by activating BAT thermogenesis and promoting WAT browning, which led to enhanced EE, as depicted in the schematic diagram in Figure 9. Moreover, we reported fat depot‐specific responses to Met/Mir treatment in thermogenesis, lipolysis, and fatty acid oxidation. In addition, Met/Mir generated measurable improvements in glucose tolerance in both prevention and treatment mouse models. To the best of our knowledge, this is the first study to investigate the combined effects of Met/Mir on the prevention and treatment of obesity. Our findings display the exciting potential of Met/Mir as a new pharmacological approach to prevent or treat obesity. In light of this, our report serves as a foundation for future clinical trials of Met/Mir therapy, as well as future reports comprising combination drug therapy as an anti‐obesity treatment regimen.

Consistent with previous reports,^19^, ^22^, ^27^ we found that Met alone reduced food intake, while Mir did not. Interestingly, however, Met's appetite‐suppressing effect was blocked in the presence of Mir via unknown mechanisms that are yet to be determined. One possible explanation could be that Met/Mir‐treated mice may need to increase food intake to compensate for an increase in EE. Nevertheless, this finding highlights the complex interactions between Met and Mir in controlling food intake and further investigation is warranted. Our data also confirm previous reports that Met increases EE in DIO mice^22^ but not in HFD‐fed mice^19^ and that Mir increases EE in DIO mice.^27^ By combining Met and Mir, EE was boosted to greater levels than either drug alone, particularly in the treatment model. This is the first evidence of how Met/Mir therapy produces additive properties to change EI and EE that neither drug could achieve alone.

Some studies have reported that both Met and Mir can individually activate BAT thermogenesis,^27^, ^28^, ^29^, ^39^, ^40^ while others have shown that only Mir but not Met promotes BAT thermogenesis.^39^, ^41^, ^42^ Our study was consistent with the latter, finding that Mir but not Met enhanced BAT thermogenesis in both mouse models. The exact reasons for the discrepancies in Met's thermogenic effects in the literature are not clear, but it could be due to the differences in mouse strain and age, diet composition, treatment regimen, and drug dosage in various studies. Of particular importance, an additive augmentation of BAT thermogenesis was produced by Met/Mir in the treatment model, which could be responsible for the greater level of overall EE. Furthermore, an increase in browning of WATs by Met/Mir treatment also contributed to this greater EE, leading to lower body adiposity in the treatment model. This was an intriguing finding given that Met alone did not significantly promote browning of scWAT in HFD‐fed mice in either treatment or prevention models, consistent with the literature.^41^ In addition, Mir has been found to augment scWAT browning in chow‐fed^40^ and DIO^31^ mice, and it was also observed to increase WAT browning in humans with obesity^43^, ^44^ but interestingly, not in healthy individuals.^28^ It appears that Mir's thermogenic effects are dependent on adiposity or metabolic contexts, which may provide an explanation for our observation that Met/Mir did not produce an additive increase in scWAT browning in the prevention model, as there was not sufficient adiposity for Met/Mir to have an additive effect. This was also the case for BAT thermogenesis, suggesting that metabolic conditions could alter the action of Met/Mir, underpinning a complex pharmacodynamic interaction that requires further elucidation.

We are the first to report this depot‐specific expression profile of lipolysis in response to Met and/or Mir treatments under two distinct clinical contexts. As lipolysis provides substrates required for BAT thermogenesis and free fatty acid is a direct stimulator of UCP1 activity, BAT lipolysis is expected to be correlated with BAT activity. This is the case in our prevention model where increased BAT lipolysis by Met/Mir treatment was coupled with increased BAT thermogenesis. Interestingly, however, differential effects of Met, Mir, and Met/Mir on lipolysis in other adipose depots were identified. For example, we found that in scWAT, Mir alone increased lipolysis,^29^, ^40^ whereas Met suppressed it,^32^ consistent with previous studies. However, when given as combination therapy, the opposing actions of these two drugs were cancelled out, leading to no change in lipolysis in scWAT. Lipolysis in eWAT was further differentially regulated, exhibiting either an increase or no change in lipolysis from Met/Mir treatment. The differential expression patterns in lipolysis highlight the complex responses of different adipose depots toward drug treatments under different metabolic settings, an area that has not been previously studied and requires further investigation.

This study confirmed that both Met and Mir monotherapy improve glucose tolerance, yet no further enhancement was observed under combined Met/Mir treatment in either of our models. Noticeably, however, Met/Mir additively improved insulin responsiveness under obesogenic conditions, contributing to the protective effect against HFD‐induced weight gain in the prevention model. This superior improvement in insulin responsiveness compared to monotherapy may be attributed to the greater reduction in adiposity that Met/Mir produced, subsequently resulting in improved glucose homeostasis. However, Met/Mir may also have other mechanisms to modulate insulin responsiveness that may explain this additive effect. Met has been found to improve insulin sensitivity via the AMP‐activated protein kinase pathway.^16^ It is unknown whether Mir similarly acts on this pathway; however, Mir is believed to indirectly stimulate insulin secretion from pancreatic β‐cells by activating lipolysis.^43^ Additionally, in recent years, Met has been found to modulate the gut microbiome, resulting in improved glucose homeostasis in HFD‐fed mice.^45^ A new clinical trial (https://clinicaltrials.gov/show/NCT04766021) aims to investigate the association between the gut microbiome and Mir metabolism. The exact mechanism by which Met/Mir improves glucose homeostasis remains an interesting new topic warranting future investigation.

Considering that the majority of anti‐obesity pharmacotherapies failed to reach the market due to unwanted side effects as opposed to poor efficacy, it was crucial to evaluate the potential adverse effects of Met/Mir therapy. Mir has been associated with adverse cardiovascular effects^46^; however, multiple human clinical trials have reported good tolerability of Mir at different doses and treatment durations and among different populations, without severe cardiovascular effects.^28^, ^29^, ^43^ Met is widely prescribed and well tolerated at higher doses. By administering 250 mg/kg BW of Met to the mice, we produced analogous plasma concentrations to those seen in clinical settings, making it a clinically relevant dose unlikely to cause significant adverse effects.^19^ Our study found that Met/Mir causes no apparent adverse effects on the internal viscera, notably showing no cardiovascular side effects. However, our study does not comprehensively evaluate whether long‐term treatment produces adverse effects. This is an important area to address in future studies given that obesity is a chronic disease requiring long courses of treatments. Furthermore, safety in human subjects will need to be carefully assessed in clinical trials before Met/Mir treatment can be offered to individuals with obesity.

## 4 CONCLUSION

This study is the first to demonstrate that a combination of Met and Mir has an additive effect on preventing and treating obesity, with measurable improvements in glucose homeostasis and minimal adverse effects. Our findings provide a novel route to managing obesity where both EI and EE can be modulated using pre‐existing drugs. From a clinical perspective, this research will provide critical preclinical evidence for future clinical trials in humans.

## 5 MATERIALS AND METHODS

### 5.1 Animals

Male 8‐week‐old C57BL/6J mice were purchased from Vital River Laboratory Animal Technology Co., Ltd. (Beijing, China). Mice were housed in a specific pathogen‐free animal facility at a standard 12‐h light/12‐h dark cycle with ad libitum access to water and food unless stated otherwise. Mice were acclimatized to the environment for 1 week before the experiments.

### 5.2 Drug treatment and BW measurement in the prevention regimen

Forty‐eight mice (BW, mean ± SD, 26.8 ± 0.6 g) were divided into four groups based on BW. They were fed a HFD (60% calories from fat, 20% calories from carbohydrate, and 20% calories from protein, SYSE, China) and concurrently received daily gavage with either Met (250 mg/kg/day, Aladdin), Mir (10 mg/kg/day, Meryer), Met/Mir (250/10 mg/kg/day) or Veh at 5:00 p.m. for 12 weeks. The chosen dosages of Met and Mir were based on the previous literature.^19^, ^27^ All drugs were dissolved or suspended in the Veh, 0.4% (w/v) carboxymethylcellulose sodium solution (CMC‐Na, Macklin). BW was monitored weekly at a fixed time of the day throughout the experimental period, and BW change was calculated as follows: (BW – BW_initial_)/BW_initial_ × 100%.^47^

### 5.3 Drug treatment and BW measurement in the treatment regimen

Mice (BW, mean ± SD, 26.8 ± 0.6 g) were first fed a HFD for 9 weeks to establish a DIO phenotype. Then, 56 DIO mice with an average BW of 41.1 ± 2.3 g (mean ± SD) were divided into four groups based on BW and received daily oral gavage with Veh, Met, Mir, or Met/Mir as described above for five continuous weeks. BW was monitored daily at a fixed time of the day, and BW change was calculated as described above.

### 5.4 Measurement of food intake and water intake

Spontaneous food and water intake were measured after the mice had acclimatized to a single cage for 24 h with ad libitum access to water and food.^48^, ^49^ At the start of measurement, equal amounts of food and water were given. The remnants of food and water were measured 24 h later. Food spillages on the cage floor were also carefully collected and weighed. Food and water intake were calculated as the amount given minus the amounts of remnants and spillages.

### 5.5 Intraperitoneal glucose tolerance test and insulin tolerance test

Intraperitoneal glucose tolerance tests (IPGTTs) and insulin tolerance tests (ITTs) were performed as previously reported.^22^ Briefly, mice were injected intraperitoneally with a glucose solution (2.0 g/kg BW; Chemical Reagent) after overnight fasting for IPGTT or insulin (0.75 U/kg BW; Eli Lilly) after 6 h of fasting for ITT. Blood glucose levels were measured at the time points indicated using a digital glucometer.

### 5.6 Determination of EE by indirect calorimetry

Mice were acclimatized to Promethion Metabolic Systems (Sable Systems International, USA) for 24 h. Then, oxygen consumption, CO_2_ release, and ambulatory activity were monitored for 24 h as previously described.^50^, ^51^ Hourly data for a period of 12 and 24 h immediately after gavage were also presented to demonstrate an acute response to drug treatments.

### 5.7 Drug treatment and metabolic characterization in pair‐fed mice

A cohort of 24 male mice (10 weeks of age, BW, mean ± standard deviation [SD], 25.3 ± 1.6 g) was divided into four groups and single housed. The mice received the same amount of HFD based on the food intake of the Met‐treated animals on the previous day, and concurrently received daily gavage of Veh, Met, Mir, or Met/Mir for 2 weeks. Blood glucose levels were measured at 9:00 a.m., both before and after drug administration with HFD pair‐feeding. Mice were fasted for 6 h before performing IPGTT and ITT at 8 and 10 days, respectively, after the commencement of treatments.^52^, ^53^ The energy metabolism of HFD pair‐feeding mice was determined as described above after drug treatment.

### 5.8 Analysis of body composition

The body composition of mice was determined using an animal NMR system (MesoQMR23‐060H‐I, China) at the end of the experiment.

### 5.9 RNA extraction and quantitative real‐time PCR analysis

The total RNA of adipose tissues was extracted by TRIzol (Life, USA). RNA (1000 ng) was reverse‐transcribed using a Primerscript RT Reagent Kit (Takara). Real‐time PCR was performed using TB Green Premix Ex Taq II (Takara). The CT value of each gene product was normalized to the housekeeping gene 18S ribosomal RNA (Rn18s).^54^ The primer sequences are listed in Table S1.

### 5.10 Western blot analysis

Proteins were extracted from adipose tissue by using RIPA lysis buffer containing a protease/phosphatase inhibitor cocktail (Roche). Western blotting was performed following a standard protocol. The antibodies are shown in Table S2. Immunoblotted bands were detected using enhanced chemiluminescence reagents, imaged by a UVP ChemStudio Imaging system (Analytik Jena), and quantified by densitometry using Fiji software (NIH).

### 5.11 Statistics

Statistical analyses were performed using GraphPad Prism 9.0 software (Chicago, IL, USA) based on the design of each experiment and are indicated in each figure legend. The data are presented as mean ± standard error of mean. ns, not significant, ^*^ *p* < 0.05, ^**^ *p* < 0.01, and ^***^ *p* < 0.001.

## AUTHOR CONTRIBUTIONS

Q.P.W. and X.Y.Z. conceived and designed the project, drafted the original manuscript, and reviewed and edited the manuscript. X.Y.Z. and Y.L. performed the majority of experiments and analyzed and interpreted the data. X.Z. contributed to data analysis and interpretation, conducted pair‐feeding‐related experiments, and revised and edited the manuscript. B.C.Z., Z.C.Y., Y.L., A.Q.L., R.X.Z., and Z.Y.L. performed part of the animal work and molecular biology experiments. G.B. reviewed and edited the manuscript. Y.C.S. provided critical guidance for the experimental design, interpreted data, acquired funding, and reviewed, revised, and finalized the manuscript. Q.P.W. oversaw the project, acquired funding, and finalized the manuscript. All authors read and approved the final manuscript.

## CONFLICT OF INTEREST

The authors declare they have no conflicts of interest.

## ETHICS STATEMENT

All animal procedures were performed following standards approved by the Sun Yat‐sen University Committee on Ethics for the Use of Laboratory Animals following the Animal Welfare Legislation of China (Ethics No. SYSU‐IACUC‐2020‐000165) and the Garvan Institute/St. Vincent's Hospital Animal Ethics Committee (Ethics #19_25), Australia.

## Supporting information

Supporting Information

## Data Availability

All the data are available from corresponding authors upon reasonable request.
