# Supplementary material for: The combined effect of metformin and mirabegron on diet‐induced obesity
Source: MedComm (2020). 2023 Feb 14;4(2):e207. doi: 10.1002/mco2.207 (PMC9928947; doi:10.1002/mco2.207)
Supplement: Supplementary file 1 — Supporting Information [file MCO2-4-e207-s001.doc]

**The Combined Effect of Metformin and Mirabegron on Diet-induced Obesity**

Running title: Metformin/mirabegron in obesity

Xin-Yuan Zhao^1,#^, Ying Liu^1,#^, Xuan Zhang^2,#^, Ben-Chi Zhao^1^, George Burley^2^, Zhi-Can Yang^1^, Yi Luo^1^, An-Qi Li^1^, Ruo-Xin Zhang^1^, Zhi-Ying Liu^1^, Yan-Chuan Shi^2,3,*^, Qiao-Ping Wang^1,*^

1 Lab of Metabolism and Aging, School of Pharmaceutical Sciences (Shenzhen), Shenzhen Campus of Sun Yat-Sen University, Sun Yat-Sen University, Shenzhen, 518107, China

2 Obesity and Metabolic Disease Research Group, Diabetes and Metabolism Division, Garvan Institute of Medical Research, Darlinghurst, 2010, Sydney, Australia

3 School of Clinical Medicine, St Vincent’s Clinical Campus, Faculty of Medicine and Health, UNSW Sydney, Australia

# These authors contributed equally to this work.

*Corresponding author

E-mail address: y.shi@garvan.org.au; ORCID: 0000-0002-8368-6735

E-mail address: wangqp7@mail.sysu.edu.cn; ORCID: 0000-0003-2809-6457

**Materials and methods**

**1. Determination of** **energy expenditure by indirect calorimetry**

After oxygen consumption (VO_2_), carbon dioxide release (VCO_2_) was monitored, and respiration exchange ratio (RER) was calculated as the quotient of VCO_2_/VO_2_, with 100% carbohydrate oxidation giving a value of 1.0 and pure fat oxidation giving a value of 0.7.^1^ Energy expenditure (EE, kcal heat produced) was calculated as caloric value (CV) x VO_2_, where CV is 3.815 + 1.232 x RER.^2^ EE was normalized to BW according to the previous report.^3^ Data for the 24 h monitoring period was presented. Hourly data for a period of 12 h and 24 h immediately after gavage were also presented to demonstrate an acute response to drug treatments.

**2. Measurement of heart rate and blood pressure**

Heart rate and arterial blood pressure were measured by a non-invasive blood pressure measurement system with the biological signal acquisition and analysis system (BP-2010A, China). Briefly, the mouse was restrained on a fixed frame and placed on a 37 °C heating pad. After acclimation, the sensor was placed on the root of the mouse’s tail. The heart rate, SBP, DBP and MBP were measured in real-time. Three rounds of measurement were conducted and averaged for an accurate reading.

**3.** **Tissue collection**

Mice were anesthetized by intraperitoneal injection of pentobarbital sodium (50 mg/kg) and then euthanized to collect blood and various tissues. The dissected tissues were weighed, snap-frozen in liquid nitrogen and stored at -80 ℃ for later use.

**4.** **Hepatic glycogen assay**

Hepatic glycogen was determined as previously reported.^4^ Briefly, the liver (20 mg) was weighed, homogenized, and dissolved in 1 M KOH solutions. After 95% ethanol extraction, the pellet was resuspended in 1 mL amyloglucosidase reaction buffer (1.4 mg/mL in 0.25 M acetate buffer, pH 4.75) and incubated in the 37 °C shaking incubator overnight. The supernatant was collected and a glucose assay was performed using Glucose Kit (Nanjing Jiancheng Bioengineering Institute) according to the manufacturer’s instructions.


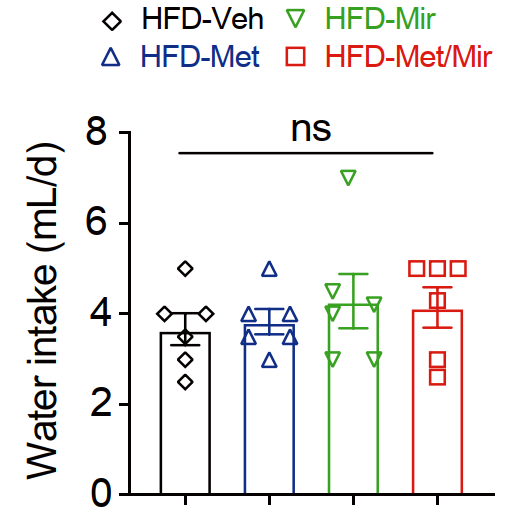


**Figure** **S1. Met/Mir does not affect water intake in mice on HFD feeding.** Water intake of mice that fed on HFD and simultaneously treated with Veh, Met, Mir or Met/Mir for 12 weeks. Data are reported as mean ± S.E.M., n=6. One-way ANOVA with Tukey’s multiple comparisons test. n.s., not significant, *P < 0.05, **P < 0.01 and ***P < 0.001.


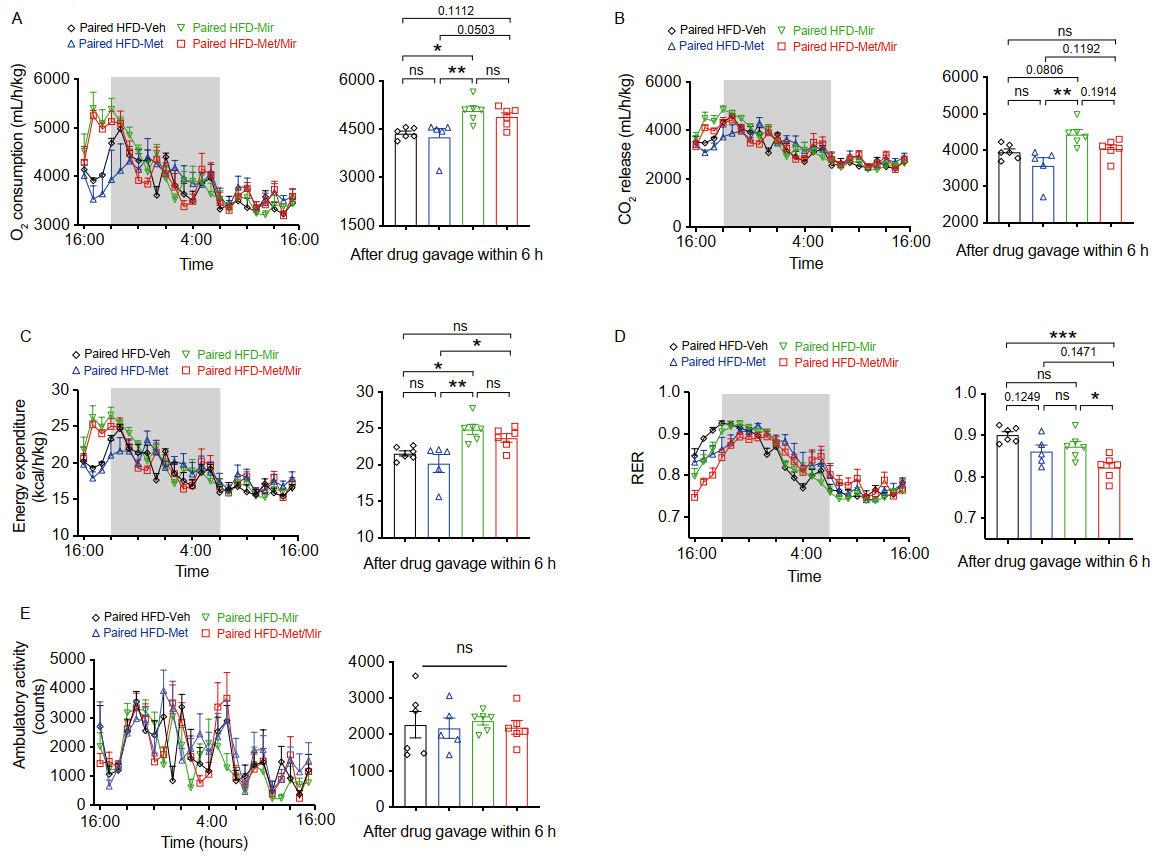


**Figure S2. Met/Mir increases EE in mice on HFD pair-feeding.** Nine-week-aged male C57BL/6J mice were pair-fed on HFD (60% fat) with controlled isocaloric intake and daily gavage administration with Veh, Met, Mir, and Met/Mir for 2 weeks. The O_2_ consumption (**A**), CO_2_ release (**B**), EE (**C**), RER (**D**), and ambulatory activity (**E**) were measured for 24 hours in mice on HFD pair-feeding and being treated with Veh, Met, Mir, and Met/Mir. Data are reported as mean ± S.E.M., n = 6 . One-way ANOVA with Tukey’s multiple comparisons test. n.s., not significant, *P < 0.05, **P < 0.01 and ***P < 0.001.


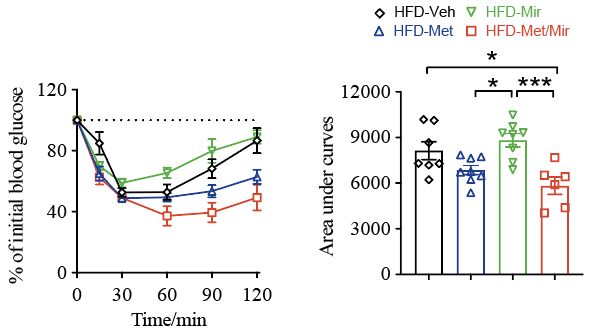


**Figure S3. Met/Mir has an additive effect on improving insulin responsiveness in HFD-fed mice.** ITT in HFD-fed mice was performed after all treatments for 12 weeks (n = 6 - 8) and the blood glucose levels were normalized to the initial blood glucose in all groups. Data were reported as mean ± S.E.M. One-way ANOVA with Tukey’s multiple comparisons test. *P < 0.05, **P < 0.01 and ***P < 0.001.


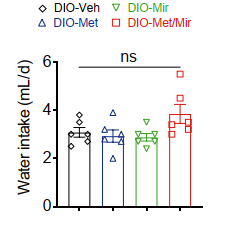


**Figure S4. Met/Mir has no effect on water intake in DIO mice.** Water intake of DIO mice after treatment with Veh, Met, Mir, or Met/Mir for 5 weeks. Data are reported as mean ± S.E.M., n = 6, one-way ANOVA with Tukey’s multiple comparisons test. n.s., not significant, *P < 0.05, **P < 0.01 and ***P < 0.001.


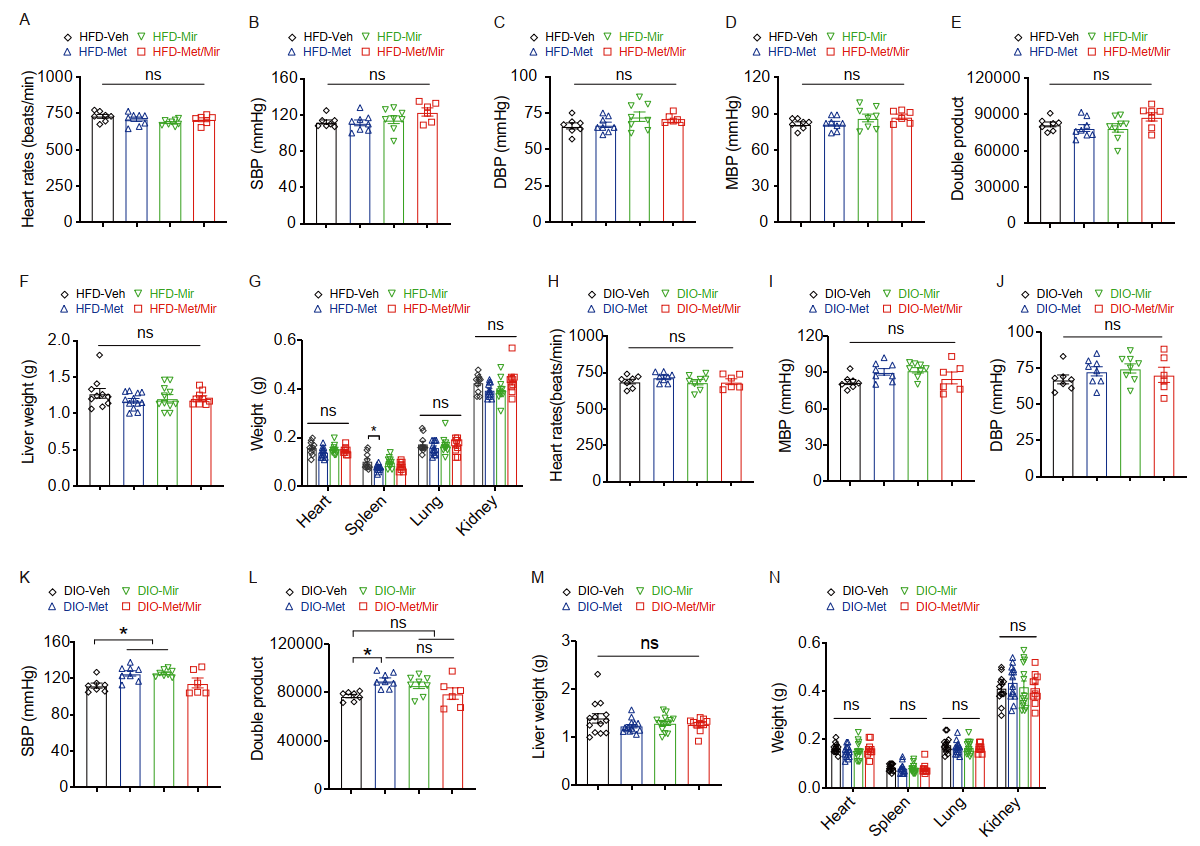


**Figure S5. Met/Mir does not affect the cardiovascular functions as well as on the weights of visceral organs in mice**. (**A - G**) Heart rates (**A**, n = 6 - 8), MBP (**B**, n = 6 - 8), DBP (**C**, n = 6 - 8), SBP (**D**, n = 6 - 8), double product (**E**, n = 6 - 8), weight of liver (**F**, n = 9 - 12) and viscera (**G**, n = 9 - 12) of mice on HFD feeding with the administration of Veh, Mir, Met, and Met/Mir for 12 weeks. (**H-N**) Heart rates (**H**, n = 6 - 8), MBP (**I**, n = 6 - 8), DBP (**J**, n = 6 - 8), SBP (**K**, n = 6 - 8), double product (**L**, n = 6 - 8), weight of liver (**M**, n = 10 - 14) and viscera (**N**, n = 10 - 14) of DIO mice with the administration of Veh, Met, Mir and Met/Mir for 5 weeks. Data are reported as mean ± S.E.M.. one-way ANOVA with Tukey’s multiple comparisons test. n.s., not significant, *P < 0.05, **P < 0.01 and ***P < 0.001.

Table S1. Primer sequences of mice in Q-PCR.

| Genes | Forward Primer | Reverse Primer |
| --- | --- | --- |
| *Rn18s* | GTAACCCGTTGAACCCCATT | GTAACCCGTTGAACCCCATT |
| *Atgl* | GGATGGCGGCATTTCAGACA | CAAAGGGTTGGGTTGGTTCAG |
| *Hsl* | CCAGCCTGAGGGCTTACTG | CTCCATTGACTGTGACATCTCG |
| *Mgl* | ACCATGCTGTGATGCTCTCTG | CAAACGCCTCGGGGATAACC |
| *Acox1* | TAACTTCCTCACTCGAAGCCA | AGTTCCATGACCCATCTCTGTC |
| *Acsl1* | TGCCAGAGCTGATTGACATTC | GGCATACCAGAAGGTGGTGAG |
| *Cpt1α* | CTCCGCCTGAGCCATGAAG | CACCAGTGATGATGCCATTCT |
| *Cpt1β* | GCACACCAGGCAGTAGCTTT | CAGGAGTTGATTCCAGACAGGTA |
| *Cpt2* | CAGCACAGCATCGTACCCA | TCCCAATGCCGTTCTCAAAAT |
| *Elovl3* | TCCGCGTTCTCATGTAGGTCT | GGACCTGATGCAACCCTATGA |
| *Ucp1* | AGGCTTCCAGTACCATTAGGT | CTGAGTGAGGCAAAGCTGATTT |
| *Dio2* | AATTATGCCTCGGAGAAGACCG | GGCAGTTGCCTAGTGAAAGGT |
| *Cidea* | TGACATTCATGGGATTGCAGAC | GGCCAGTTGTGATGACTAAGAC |
| *Pgc1α* | TATGGAGTGACATAGAGTGTGCT | CCACTTCAATCCACCCAGAAAG |
| *Prdm16* | GACATTCCAATCCCACCAGA | CACCTCTGTATCCGTCAGCA |
| *Tmem26* | TTCCTGTTGCATTCCCTGGTC | GCCGGAGAAAGCCATTTGT |
| *Cox7a1* | GCTCTGGTCCGGTCTTTTAGC | GTACTGGGAGGTCATTGTCGG |

**Table S2. Primary and secondary antibodies for Western B**lot

| **Antibodies** | **Concentration** | **Source** |
| --- | --- | --- |
| Rabbit anti-UCP1 antibody | 1:10000 | Abcam, ab209483 |
| Mice monoclonal anti-α-tubulin antibody | 1:10000 | Ray antibody, MG17 |
| Goat anti-rabbit IgG | 1:10000 | Ray antibody, RM3002 |
| Goat anti-mouse IgG | 1:10000 | Ray antibody, RM3001 |

References

1. Ferrannini E. The theoretical bases of indirect calorimetry: a review. *Metabolism*. 1988; 37(3): 287-301.

2. Yan C, Zeng T, Lee K, et al. Peripheral-specific Y1 receptor antagonism increases thermogenesis and protects against diet-induced obesity. *Nat Commun*. 2021;12(1):2622.

3. Wang Q, Li D, Cao G, et al. IL-27 signalling promotes adipocyte thermogenesis and energy expenditure. *Nature*. 2021;

4. Valsesia A, Wang QP, Gheldof N, et al. Genome-wide gene-based analyses of weight loss interventions identify a potential role for NKX6.3 in metabolism. *Nat Commun*. 2019;10(1):540.
